# Postoperative Outcomes Following Preweekend Surgery

**DOI:** 10.1001/jamanetworkopen.2024.58794

**Published:** 2025-03-04

**Authors:** Sanjana Ranganathan, Carlos Riveros, Yusuke Tsugawa, Michael Geng, Vatsala Mundra, Zachary Melchiode, Bheeshma Ravi, Natalie Coburn, Angela Jerath, Allan S. Detsky, Christopher J. D. Wallis, Raj Satkunasivam

**Affiliations:** 1 Department of Urology, Houston Methodist Hospital, Houston, Texas; 2 Division of General Internal Medicine and Health Services Research, David Geffen School of Medicine at UCLA, Los Angeles, California; 3 Department of Health Policy and Management, UCLA Fielding School of Public Health, Los Angeles, California; 4 Division of Orthopedic Surgery, Department of Surgery, Sunnybrook Health Sciences Center, Toronto, Ontario, Canada; 5 Division of Orthopedic Surgery, Department of Surgery, University of Toronto, Toronto, Ontario, Canada; 6 Division of Surgery, Department of Surgery, Sunnybrook Health Sciences Centre, University of Toronto, Ontario, Canada; 7 Department of Anesthesia, Sunnybrook Health Sciences Center, Toronto, Ontario, Canada; 8 Department of Medicine, Mount Sinai Hospital and University Health Network, Toronto, Ontario, Canada; 9 Institute for Health Policy, Management and Evaluation, University of Toronto, Toronto, Ontario, Canada; 10 Department of Medicine, University of Toronto, Toronto, Ontario, Canada; 11 Division of Urology, Department of Surgery, University of Toronto, Toronto, Ontario, Canada; 12 Division of Urology, Department of Surgery, Mount Sinai Hospital, Toronto, Ontario, Canada; 13 Department of Surgical Oncology, University Health Network, Toronto, Ontario, Canada

## Abstract

**Importance:**

The phenomenon of a *weekend effect* refers to a higher potential for adverse outcomes in patients receiving care over the weekend. Few prior studies have comprehensively investigated the effects of postoperative weekend care on surgical outcomes in a generalizable cohort.

**Objective:**

To examine differences in short-term and long-term postoperative outcomes of patients undergoing surgical procedures immediately before vs after the weekend.

**Design, Setting, and Participants:**

This is a population-based, retrospective cohort secondary analysis of adult patients in Ontario, Canada, undergoing 1 of 25 common surgical procedures between January 1, 2007, to December 31, 2019, with 1 year of follow-up. Data analysis was performed from October to November 2022.

**Exposure:**

Undergoing surgery before (1 day before) vs after (1 day after) the weekend.

**Main Outcomes and Measures:**

The primary outcome was a composite of death, readmission, and complications at 30 days, 90 days, and 1 year. Multivariable generalized estimating equations with an independent correlation structure, accounting for covariates, with clustering on surgical procedure were used to estimate the association between day of surgery in relation to the weekend and the outcomes.

**Results:**

Of the 429 691 patients (mean [SD] age, 58.6 [16.9] years; 270 002 female patients [62.8%]) in the study cohort, 199 744 (46.5%) underwent surgery before the weekend, and 229 947 (53.5%) underwent surgery after the weekend. Patients in the preweekend group were more likely than those in the postweekend group to experience the composite outcome of death, complications, and readmissions at 30 days (adjusted odds ratio [aOR], 1.05; 95% CI, 1.02-1.08), 90 days (aOR, 1.06; 95% CI, 1.03-1.09), and 1 year (aOR, 1.05; 95% CI, 1.02-1.09) after surgery. Odds of mortality were increased in the preweekend group vs the postweekend group at 30 days (aOR, 1.09; 95% CI, 1.03-1.16), 90 days (aOR, 1.10; 95% CI, 1.03-1.17), and 1 year (aOR, 1.12; 95% CI, 1.08-1.17).

**Conclusions and Relevance:**

In this retrospective multi-institutional study, patients who underwent surgery immediately preceding the weekend had a significantly increased risk of complications, readmissions, and mortality compared with those treated after the weekend. Further study is needed to understand differences in care that may underpin these observations and ensure that patients receive high-quality care regardless of the day of the week.

## Key Points

**Question:**

Is surgery immediately before vs after the weekend associated with postoperative outcomes?

**Findings:**

In a cohort study involving 429 691 patients undergoing 25 common surgical procedures in Ontario, Canada, those who underwent surgery immediately before the weekend experienced a statistically significant increase in the composite outcome of death, complications, and readmissions at 30 days, 90 days, and 1 year compared with those treated after the weekend.

**Meaning:**

These findings suggest that patients treated before the weekend are at increased risk of complications, emphasizing the need for further investigation into processes of surgical care to ensure consistent high-quality care and patient outcomes.

## Teaser

This cohort study examines 30-day, 90-day, and 1-year postoperative outcomes of patients undergoing surgical procedures immediately before vs after the weekend.

## Introduction

Hospitals and health care systems have variations in operational structure and organization during the transition from weekdays to weekends.^1,2^ The *weekend effect* refers to the potential for worse patient outcomes during the weekends, compared with weekdays.^3,4,5,6^ In surgery, this concept may also apply to those undergoing surgery immediately before the weekend, who receive postoperative care during the weekend. Several studies^7,8,9,10,11,12,13^ with a focus on mortality among specific subspecialties have demonstrated conflicting results.

To date, few studies have examined the weekend effect on postoperative outcomes comprehensively, across a variety of outcomes (eg, mortality and complications) at short-term (30 days), intermediate (90 days), and long-term (1 year) time periods among multiple surgical specialties, including both elective and emergent procedures. Thus, we performed a population-based, retrospective cohort study of patients undergoing surgery in Ontario, Canada, to examine the association between the weekend effect (comparing surgery immediately before the weekend with surgery after the weekend) and postoperative outcomes. We hypothesized that surgery performed immediately before a weekend is associated with worse postoperative outcomes compared with surgery performed after the weekend.

## Methods

### Study Setting and Design

We conducted a population-based, retrospective cohort analysis of adults undergoing common surgical procedures in Ontario, Canada, between January 1, 2007, and December 31, 2019. In Ontario, residents have access to a universal health care plan delivered by a single government payer, the Ontario Health Insurance Program. We identified representative procedures across a variety of subspecialties, including cardiothoracic surgery, general surgery, neurosurgery, obstetrics and gynecology, orthopedic surgery, otolaryngology, plastic surgery, thoracic surgery, urology, and vascular surgery using a multidisciplinary consensus process including surgeons from a number of subspecialties, anesthesiologists, and internists (not coauthors of this article).^14,15,16^ This study was performed in accordance with Strengthening the Reporting of Observational Studies in Epidemiology (STROBE) reporting guidelines.^17^ The study protocol was approved by the Mount Sinai Hospital Research Ethics Board. Informed consent was not needed because the data are deidentified, in accordance with 45 CFR §46.

### Data Sources

We retrieved data from multiple health care databases available from ICES. We gathered information regarding primary procedures and complications from records of the Ontario Health Insurance Program database; diagnostic, procedural, and discharge data from the Canadian Institute for Health Information Discharge Abstract Database and Same Day Surgery databases; patient demographic information from the Registered Persons Database; and physician demographic information from the ICES Physician Database.

### Cohort Derivation

We selected adult patients who had undergone 1 of 25 procedures of interest (eTable 1 in Supplement 1) during the study period (eFigure in Supplement 1). We excluded patients who were younger than 18 years, those who were not Ontario residents, those with missing data, those undergoing multiple surgical procedures on the same day, and those whose surgical procedures did not fall on a preweekend or postweekend day.

### Outcome Measures

The primary outcome was a binary composite of mortality, complications, and readmissions at 30 days following the procedure.^18,19^ The secondary outcomes were the composite outcome at 90 days and 1 year, along with death, any-cause readmission, and complications at each time point, as well as hospital length of stay (LOS) and duration of index surgery. All outcomes were determined a priori. Included complications are shown in eTable 2 in Supplement 1. We identified outcomes utilizing procedural and diagnostic codes collected for all patients and hospitals in Ontario.^18,19,20^

### Exposure

We assessed the patient’s date of surgery as either the day before the weekend (usually Friday or the day immediately before a long weekend) or the day after the weekend (usually Monday or the day immediately after a long weekend). We included 9 national Canadian holidays observed in Ontario: New Year’s Day, Family Day, Good Friday, Victoria Day, Canada Day, Labour Day, Thanksgiving Day, Christmas Day, and Boxing Day.

### Covariates

We captured covariates related to patient, surgeon, anesthesiologist, facility, and treatment characteristics. Patient variables included age, sex, comorbidity burden (Johns Hopkins Aggregate Diagnosis Group), rurality, and socioeconomic status. Physician and anesthesiologist variables included age, sex, specialty, annual case volume, and years in practice. Facility and treatment variables included facility type (academic vs community), admission route (elective vs urgent), case complexity, as well as year and duration of index surgery (eTable 3 in Supplement 1).

### Statistical Analysis

Data analysis was performed from October to November 2022. We compared the patient, surgeon, anesthesiologist, facility, and treatment characteristics between the preweekend and postweekend groups using standardized differences, with a standardized difference defined as greater than 0.10.^21^

We utilized multivariable generalized estimating equations with an independent correlation structure, accounting for covariates, with clustering on surgical procedure to estimate the association between day of surgery in relation to the weekend and outcomes at 30 days, 90 days, and 1 year from the index surgery. Given the between-procedure variation in outcomes, we elected to cluster on the procedure performed; thus, the model allows us to functionally compare outcomes within a procedure (eTable 4 in Supplement 1).

To estimate adjusted absolute event rates and means, we used models with a Poisson distribution with log link for binary outcomes and models with negative binomial with log link for continuous outcomes (LOS and duration of surgery). The risk-adjusted absolute difference and 95% CI were calculated for the composite outcome at each time point. Models with logit link were used to estimate adjusted relative effects (presented as adjusted odds ratio [aOR] for binary outcomes or adjusted relative risk for continuous outcomes). Estimates were adjusted for the median value of continuous covariates and the third quartile or quintile of categorical covariates. We performed a priori sensitivity analysis by adding duration of the index surgery as an additional covariate.

We performed subgroup analyses based on predetermined variables to assess for heterogeneity of effect according to patient, surgeon, anesthesiologist, facility, and treatment characteristics. To further evaluate heterogeneity of effect, we evaluated several a priori determined subgroups, including surgical urgency (emergent vs elective) and case complexity (low vs high) (eTable 5 in Supplement 1).^15,20^ To ensure that the types of surgery done before and after the weekend did not impact outcomes, we used clustering based on procedure fee codes such that, analytically, outcomes of patients were only compared within the same procedure.^14,20^

We performed several post hoc sensitivity analyses. To investigate a dose-response association between proximity to the weekend and outcomes, we included patients undergoing surgery 2 days before (usually Thursday and Friday) and after (usually Monday and Tuesday) the weekend. Next, among emergent cases, we examined whether deferral of surgery (eg, delay of treatment over the weekend) affected outcomes. We defined deferred emergency surgery as those undergoing emergent surgery 2 or more days after the same emergent admission for the index surgery. We eliminated holiday weekends from our analysis, limiting the cohort to patients undergoing surgery surrounding regular weekends. To assess for potential differences in associations within a modern subgroup, we limited the cohort to patients undergoing surgery from 2015 to 2019. Finally, we limited the analytic cohort to patients spending at least 48 hours in the hospital following surgery.

Statistical significance was set at *P* < .05 based on a 2-tailed comparison. All analyses were performed using SAS Enterprise Guide software version 6.1 (SAS Institute).

## Results

### Baseline Demographics and Outcomes

The final cohort included 429 691 patients (mean [SD] age, 58.6 [16.9] years; 270 002 female patients [62.8%]); 199 744 (46.5%) were in the preweekend group, and 229 947 (53.5%) were in the postweekend group (Table 1). Most of the patients lived in urban areas (379 056 patients [88.2%]), with an approximately even distribution of the patients across income quintile groups (Table 1). Of the studied procedures, 363 608 (84.6%) were elective and 66 083 (15.4%) were urgent. The only characteristics with a standardized difference exceeding 0.10 were surgeon age and years in practice, with surgeons in the preweekend group being slightly younger (median [IQR] age, 47 [40-55] years vs 48 [42-56] years) and less experienced (median time in practice, 14 [7-22] years vs 17 [8-23] years) than surgeons in the postweekend group (Table 1). All other patient, surgeon, anesthesiologist, and hospital characteristics were balanced. Surgeons operating on Friday had slightly fewer years in practice (mean [SD], 22.5 [10.2] years) compared with the average across all weekdays (mean [SD], 23.1 [10.0] years) (eTable 6 in Supplement 1).

**Table 1.** Baseline Characteristics of Study Cohort

Variable	Participants, No. (%)			Standardized difference
	Preweekend surgery (n = 199 744)	Postweekend surgery (n = 229 947)	Total (N = 429 691)	
Patient characteristics				
Age, y				
Mean (SD)	58.5 (17.1)	58.8 (16.6)	58.6 (16.9)	0.018
Median (IQR)	60 (46-71)	60 (47-71)	60 (47-71)	0.017
Sex				
Female	124 554 (62.4)	145 448 (63.3)	270 002 (62.8)	0.019
Male	75 190 (37.6)	84 499 (36.7)	159 689 (37.2)	0.019
Comorbidity, Johns Hopkins Aggregate Diagnosis Group				
0-5	51 119 (25.6)	61 024 (26.5)	112 143 (26.1)	0.022
6-7	48 263 (24.2)	56 011 (24.4)	104 274 (24.3)	0.005
8-10	60 481 (30.3)	69 010 (30.0)	129 491 (30.1)	0.006
≥11	39 881 (20.0)	43 902 (19.1)	83 783 (19.5)	0.022
Rurality				
Urban	177 250 (88.7)	201 806 (87.8)	379 056 (88.2)	0.030
Rural	22 494 (11.3)	28 141 (12.2)	50 635 (11.8)	0.030
Income quintile				
1, Lowest	38 489 (19.3)	43 113 (18.7)	81 602 (19.0)	0.013
2	40 416 (20.2)	46 386 (20.2)	86 802 (20.2)	0.002
3	40 413 (20.2)	46 138 (20.1)	86 551 (20.1)	0.004
4	40 655 (20.4)	47 228 (20.5)	87 883 (20.5)	0.005
5, Highest	39 771 (19.9)	47 082 (20.5)	86 853 (20.2)	0.014
Surgeon characteristics				
Age, y				
Mean (SD)	48.1 (9.6)	49.1 (9.3)	48.6 (9.5)	0.096
Median (IQR)	47 (40-55)	48 (42-56)	48 (41-56)	0.104
Sex				
Female	29 658 (14.8)	32 478 (14.1)	62 136 (14.5)	0.021
Male	170 086 (85.2)	197 469 (85.9)	367 555 (85.5)	0.021
Annual case volume (quartiles)				
1, Lowest	49 320 (24.7)	52 242 (22.7)	101 562 (23.6)	0.046
2	50 503 (25.3)	59 878 (26.0)	110 381 (25.7)	0.017
3	48 463 (24.3)	59 815 (26.0)	108 278 (25.2)	0.040
4, Highest	51 458 (25.8)	58 012 (25.2)	109 470 (25.5)	0.012
Time in practice, y				
Mean (SD)	14.6 (8.7)	15.9 (8.5)	15.3 (8.6)	0.145
Median (IQR)	14 (7-22)	17 (8-23)	16 (8-22)	0.149
Specialty				
Cardiothoracic surgery	545 (0.3)	932 (0.4)	1477 (0.3)	0.023
General surgery	67 857 (34.0)	69 298 (30.1)	137 155 (31.9)	0.082
Neurosurgery	10 506 (5.3)	11 848 (5.2)	22 354 (5.2)	0.005
Obstetrics and gynecology	26 887 (13.5)	33 272 (14.5)	60 159 (14.0)	0.029
Orthopedic surgery	63 891 (32.0)	79 125 (34.4)	143 016 (33.3)	0.051
Otolaryngology	3484 (1.7)	4492 (2.0)	7976 (1.9)	0.016
Plastic surgery	8756 (4.4)	9563 (4.2)	18 319 (4.3)	0.011
Thoracic surgery	2480 (1.2)	3338 (1.5)	5818 (1.4)	0.018
Urology	14 701 (7.4)	17 375 (7.6)	32 076 (7.5)	0.007
Vascular surgery	637 (0.3)	704 (0.3)	1341 (0.3)	0.002
Anesthesiologist characteristics				
Age, y				
Mean (SD)	48.9 (10.2)	49.0 (10.1)	49.0 (10.2)	0.005
Median (IQR)	48 (41-57)	48 (41-57)	48 (41-57)	0.008
Sex				
Female	51 887 (26.0)	61 747 (26.9)	113 634 (26.4)	0.020
Male	147 857 (74.0)	168 200 (73.1)	316 057 (73.6)	0.020
Annual case volume (quartiles)				
1, Lowest	45 761 (22.9)	53 869 (23.4)	99 630 (23.2)	0.012
2	52 586 (26.3)	59 221 (25.8)	111 807 (26.0)	0.013
3	51 178 (25.6)	57 695 (25.1)	108 873 (25.3)	0.012
4, Highest	50 219 (25.1)	59 162 (25.7)	109 381 (25.5)	0.013
Time in practice, y				
Mean (SD)	14.6 (9.5)	14.7 (9.4)	14.7 (9.4)	0.005
Median (IQR)	14 (6-23)	14 (6-22)	14 (6-23)	0.007
Other characteristics				
Hospital status				
Community hospital	131 102 (65.6)	149 787 (65.1)	280 889 (65.4)	0.010
Academic hospital	68 642 (34.4)	80 160 (34.9)	148 802 (34.6)	0.010
Surgical procedure type				
Elective	165 397 (82.8)	198 211 (86.2)	363 608 (84.6)	0.094
Urgent	34 347 (17.2)	31 736 (13.8)	66 083 (15.4)	0.094
Case complexity				
Low	69 728 (34.9)	73 206 (31.8)	142 934 (33.3)	0.065
High	130 016 (65.1)	156 741 (68.2)	286 757 (66.7)	0.065
Duration of index surgery				
Data missing	11 242 (5.6)	12 283 (5.3)	23 525 (5.5)	0.013
Nonmissing data	188 502 (94.4)	217 664 (94.7)	406 166 (94.5)	0.013
Mean (SD), min	125.1 (115.5)	123.9 (89.6)	124.5 (102.4)	0.011
Median (IQR), min	105 (75-150)	106 (78-147)	106 (76-149)	0.015
Year of index surgery				
2007	16 835 (8.4)	18 958 (8.2)	35 793 (8.3)	0.007
2008	16 564 (8.3)	19 300 (8.4)	35 864 (8.3)	0.004
2009	16 658 (8.3)	19 676 (8.6)	36 334 (8.5)	0.008
2010	16 468 (8.2)	19 273 (8.4)	35 741 (8.3)	0.005
2011	16 481 (8.3)	18 890 (8.2)	35 371 (8.2)	0.001
2012	16 365 (8.2)	18 174 (7.9)	34 539 (8.0)	0.011
2013	17 039 (8.5)	18 907 (8.2)	35 946 (8.4)	0.011
2014	16 781 (8.4)	18 950 (8.2)	35 731 (8.3)	0.006
2015	16 356 (8.2)	18 323 (8.0)	34 679 (8.1)	0.008
2016	14 584 (7.3)	17 379 (7.6)	31 963 (7.4)	0.010
2017	13 366 (6.7)	15 321 (6.7)	28 687 (6.7)	0.001
2018	11 735 (5.9)	13 975 (6.1)	25 710 (6.0)	0.009
2019	10 512 (5.3)	12 821 (5.6)	23 333 (5.4)	0.014

### Outcomes at 30 Days

At 30 days following the index surgery, patients in the preweekend group were more likely than those in the postweekend group to experience the composite primary outcome (8.49% [95% CI, 7.61%-9.46%] vs 8.13% [95% CI, 7.27%-9.10%]; aOR, 1.05 [95% CI, 1.02-1.08]) (Table 2 and Table 3). The risk-adjusted absolute difference was 0.36% (95% CI, 0.21%-0.49%) (eTable 7 in Supplement 1). Furthermore, an increase was seen in all components of this composite measure (Figure). Odds of mortality were increased in the preweekend group at 30 days (aOR, 1.09 [95% CI, 1.03-1.16]). In addition, preweekend surgery was associated with a longer hospital LOS (adjusted relative risk, 1.06 [95% CI, 1.04-1.08]).

**Table 2.** Adjusted Event Rate or Mean of Outcomes Within 30 and 90 Days and 1 Year of Index Surgery^a^

Time period and outcome	Adjusted rate, % (95% CI)	
	Preweekend surgery	Postweekend surgery
Within 30 d		
Composite end point	8.49 (7.61-9.46)	8.13 (7.27-9.10)
Death	0.16 (0.10-0.25)	0.15 (0.09-0.24)
Readmission	4.31 (3.94-4.73)	4.17 (3.82-4.55)
Complications	4.25 (3.60-5.01)	4.07 (3.42-4.83)
Hospital stay, mean (95% CI), d	3.49 (3.18-3.82)	3.29 (3.01-3.58)
Duration of index surgery, mean (95% CI), min	144.73 (131.21-159.65)	143.84 (130.54-158.50)
Within 90 d		
Composite end point	12.14 (11.28-13.06)	11.58 (10.76-12.45)
Death	0.38 (0.25-0.57)	0.35 (0.23-0.54)
Readmission	7.85 (7.19-8.56)	7.50 (6.89-8.17)
Complications	4.55 (3.90-5.32)	4.36 (3.71-5.14)
Hospital stay, mean (95% CI), d	4.17 (3.80-4.58)	3.89 (3.57-4.24)
Within 1 y		
Composite end point	22.64 (21.04-24.38)	21.84 (20.49-23.29)
Death	1.44 (1.02-2.03)	1.30 (0.91-1.87)
Readmission	18.10 (16.22-20.21)	17.58 (15.89-19.44)
Complications	5.81 (5.18-6.52)	5.57 (4.94-6.29)
Hospital stay, mean (95% CI), d	5.70 (5.19-6.27)	5.32 (4.89-5.79)

^a^ Rates were calculated using generalized estimating equation modeling dealing with clustering based on procedure fee code (Poisson distribution with log link for binary outcomes and negative binominal with log link for continuous outcomes), adjusted for surgeon age (using the median age), surgeon annual case volume (using third quartile), surgeon years of practice (using the median value), anesthesiologist age (using the median age), anesthesiologist annual case volume (using third quartile), anesthesiologist years of practice (using the median value), patient age (using the median age), patient comorbidity (using Johns Hopkins Aggregate Diagnosis Group 8-10), rurality (using urban), income quintile (using third quintile), and hospital status (using academic).

**Table 3.** Sensitivity Analyses of Models Using the Composite End Point as the Outcome^a^

Model	Friday vs Monday, aOR (95% CI)		
	Outcome within 30 d^b^	Outcome within 90 d^b^	Outcome within 1 y^b^
1, Surgery performed 1 d before or after regular (nonholiday) weekends	1.06 (1.02-1.09)	1.07 (1.04-1.10)	1.06 (1.02-1.09)
2, Surgery performed from 2015-2019	1.07 (1.03-1.11)	1.06 (1.03-1.10)	1.06 (1.03-1.10)
3, Surgery performed on patients spending at least 48 h in the hospital following surgery	1.06 (1.03-1.09)	1.07 (1.04-1.10)	1.06 (1.03-1.09)

Abbreviation: aOR, adjusted odds ratio.

^a^ Analyses used generalized estimating equation modeling dealing with clustering based on procedure fee code (logistic regression with binomial distribution and logit link for binary outcomes, and negative binomial distribution and log link for continuous outcomes), adjusted for surgeon age (continuous), surgeon sex, surgeon annual case volume (quartiles), surgeon specialty, surgeon years of practice (continuous), anesthesiologist age (continuous), anesthesiologist sex, anesthesiologist annual case volume (quartiles), anesthesiologist years of practice (continuous), patient age (continuous), patient sex, patient comorbidity (categorical), rurality (rural vs urban), income quintile, local health integration network, hospital status (academic vs community), and index year.

^b^ All *P* < .001.

**Figure.**
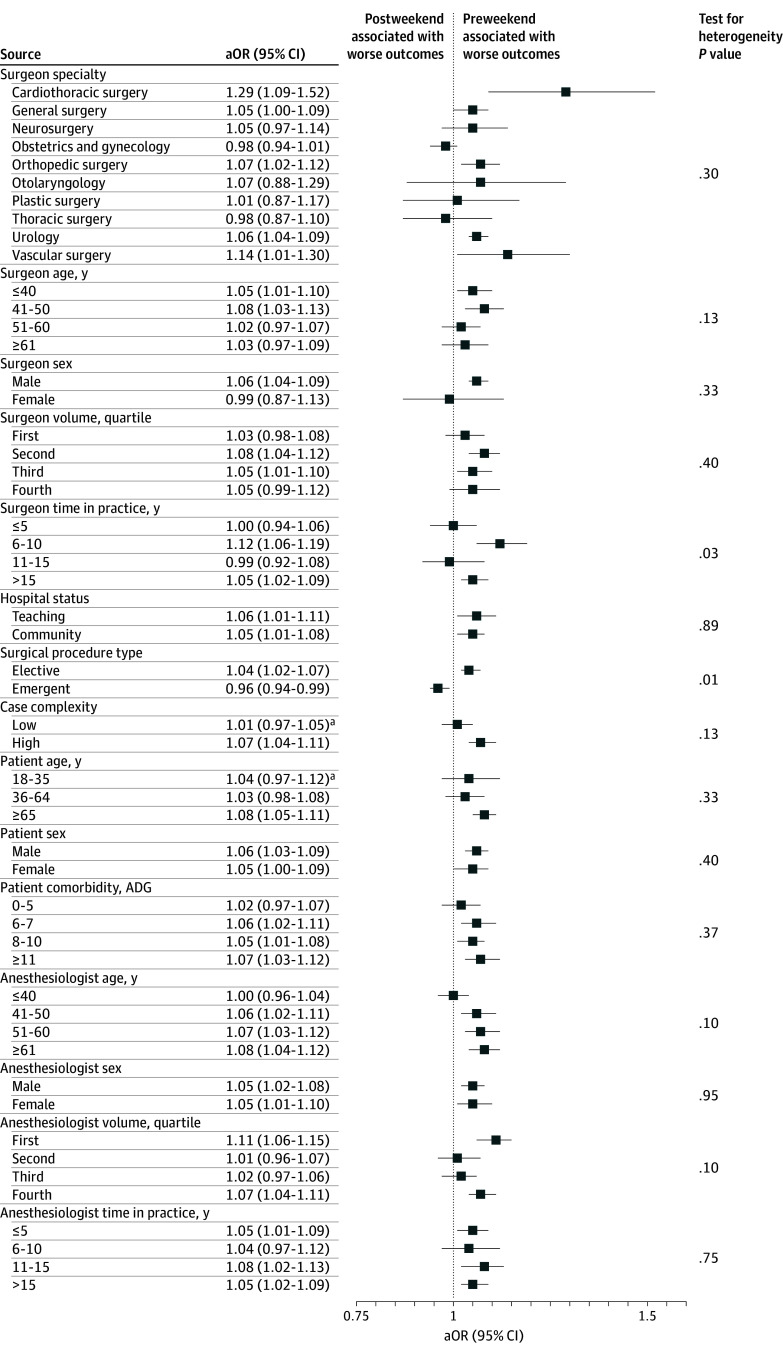
Forest Plot of Subgroup Analysis Assessing Association Between Preweekend and Postweekend Surgery, Stratified by Surgeon, Patient, Facility, Treatment, and Anesthesiologist Characteristics ADG indicates Johns Hopkins Aggregate Diagnosis Group; aOR, adjusted odds ratio.

### Intermediate (90-Day) and Long-Term (1-Year) Outcomes

Patients undergoing surgery in the preweekend group had higher rates of the composite primary outcome vs the postweekend group at 90 days (12.14% [95% CI, 11.28%-13.06%] vs 11.58% [95% CI, 10.76%-12.45]; aOR, 1.06 [95% CI, 1.03-1.09]) and at 1 year (22.64% [95% CI, 21.04%-24.38%] vs 21.84% [95% CI, 20.49%-23.29%]; aOR, 1.05 [95% CI, 1.02-1.09]) (Table 2 and Table 3). The risk-adjusted absolute difference was 0.57% (95% CI, 0.39%-0.74%) at 90 days and 0.81% (95% CI, 0.58%-1.04%) at 1 year (eTable 7 in Supplement 1). This difference was seen for each component of the primary end point (death, readmission, and complication), as well as for LOS when evaluated at 90 days and at 1 year (Figure). The odds of mortality were increased for the preweekend group vs the postweekend group at 90 days (aOR, 1.10 [95% CI, 1.03-1.17]) and 1 year (aOR, 1.12 [95% CI, 1.08-1.17]).

### Subgroup and Sensitivity Analyses

We found no evidence of effect modification when stratified by patient-specific, surgeon-specific, and anesthesiologist-specific factors, or by hospital status (Figure). However, we identified differences in outcomes by procedural urgency (elective vs emergent). Undergoing surgery before the weekend was associated with poor outcomes for elective procedures (aOR, 1.04; 95% CI, 1.02-1.07), but improved outcomes for emergent procedures (aOR, 0.96; 95% CI, 0.94-0.99) (*P* for heterogeneity = .01) (Figure). However, after removal of patients whose emergent surgery was deferred until after the weekend (deferred emergency surgery), we found the adjusted event rate of the composite end point to be higher for the preweekend group (8.24%; 95% CI, 7.39%-9.19%) compared with the postweekend group (7.91%; 95% CI, 7.07%-8.84%) at both 30 days and 90 days (eTable 8 in Supplement 1).

Results were consistent with the primary analysis across sensitivity analyses. We analyzed according to duration of surgery (eTable 9 in Supplement 1); we clustered on the basis of the procedure fee code for 30-day, 90-day, and 1-year outcomes (eTable 10 in Supplement 1); we expanded the preweekend and postweekend window to 2 days (Figure; eTable 11 in Supplement 1); we compared unadjusted vs adjusted models for patient and facility factors vs adjusted for patient, facility, and physician factors (eTable 12 in Supplement 1); we restricted to patients treated around nonholiday weekends; we restricted to a more contemporaneous cohort; and we restricted to patients spending at least 48 hours in the hospital following surgery (Table 3).

## Discussion

In this cohort study, among adults undergoing surgical procedures, the odds of adverse postoperative outcomes, including death, readmission, and complications in the short and long term, were increased by 5% for patients undergoing surgery immediately preceding the weekend. This weekend effect was seen across multiple subspecialties, in particular among patients undergoing elective operations. This study is novel in that it comprehensively analyzes the weekend effect on perioperative outcomes, including all surgical specialties, encompassing both emergent and nonemergent surgical procedures, and analyzing short-term (30-day), intermediate (90-day), and long-term (1-year) outcomes.

Our study is consistent with the majority of published literature, indicating a greater risk of adverse postoperative outcomes among patients undergoing surgery before the weekend.^9,22^ Aylin et al^9^ analyzed elective surgery across English public hospitals in multiple specialties and found that patients undergoing surgery on Monday had a decreased risk of death within 30 days compared with all other days of the week. In an international study,^23^ undergoing an elective surgical procedure on Friday vs Monday was associated with up to a 20% increased risk of 30-day mortality in the Netherlands. Furthermore, preweekend surgery has been associated with decreased process measure adherence (eg, mobilization and Foley catheter removal) in a large multi-institutional North American study.^24^ In a meta-analysis^25^ with more than 8 million patients, preweekend elective procedures (Thursday and Friday) were associated with statistically significantly higher mortality vs postweekend (Monday) surgical procedures. Specifically, Thursday was associated with a 12% higher pooled odds of short-term mortality, and Friday was associated with a 24% higher pooled odds compared with Monday.^25^ Work has also been done examining whether the weekend effect exists with specific diagnoses. For example, Palmer et al^26^ showed that patients with stroke who are admitted on the weekend have an increased likelihood of not receiving urgent care and have worse outcomes vs those who are admitted over the weekdays. In another study of more than 3000 hospitals,^27^ patients who presented for gastrointestinal hemorrhage over the weekend had a higher risk of undergoing surgery and higher rates of mortality. However, by use of data from Ontario, Dubois et al^28^ found no difference in 30-day mortality for elective surgical procedures performed on Friday compared with Monday, a finding that conflicts with those of the present study. Key methodological differences likely explain this: Dubois et al^28^ included only elective procedures that required a minimum 2-day hospital stay and excluded any procedures performed after regular working hours. Furthermore, our study added important additional metrics of complications and readmissions, and specifically incorporated long weekends into our analysis. By comparison, Dubois et al^28^ examined variations in complications across days of the week and did not identify any association between the day of the week and 30-day mortality or health safety outcomes, including readmission, reoperation, or intensive care unit admission.

The ubiquity of the weekend effect across multiple hospital systems in different countries speaks to multifactorial causes that persist despite variations in health care structure. System-level factors, such as staffing differences, service availability, and obstacles with care coordination, likely play a contributing role. In a study^29^ quantifying workforce staffing, numbers for all staff members (doctors, nurses, and other clinical staff) were shown to sharply decline over the weekend. This may contribute to the observed weekend effect via a failure-to-rescue mechanism—that is, a short-staffed weekend team may be less likely to detect and act on acute complications early in their evolution, leading to a higher complication rate for patients.

In addition to reduced personnel, there are also variations in personnel expertise over the weekend. Our results demonstrate that more junior surgeons (those with fewer years of experience) are operating on Friday, compared with Monday; this difference in expertise may play a role in the observed differences in outcomes. We examined the comparative effects of adjusting for facility, patient, and physician factors in our models (eTable 12 in Supplement 1), with the effects consistent across models, but reduced in magnitude when physician factors were added, suggesting that the weekend effect is greatly associated with physician characteristics. Diminished access to more senior colleagues or consultants on Friday may further compound this issue, and has been shown to impact complications and LOS.^22^ Furthermore, weekend teams may be less familiar with the patients than the weekday team previously managing care.^29,30^ In addition, these deficiencies are likely magnified by the reduced availability of resource-intensive tests, interventional procedures, and tools, which may be otherwise available on weekdays.^2,31,32^ Patients are less likely to be discharged over the weekend, which may be related to differences in medical personnel availability, leading to delays in discharge decision-making and increasing LOS.^33^

Our subgroup analysis demonstrated differences by procedural urgency, with lower rates of adverse events for patients undergoing emergent surgery before the weekend. This finding can be explained by a delay in care for patients presenting with emergent conditions immediately before or during the weekend, as evidenced by our analysis of deferred emergency surgery. After removal of patients who presented before the weekend but who did not undergo surgery until after the weekend, we found that the event rate of the composite end point was higher in the preweekend group, consistent with our findings across other subgroups. Immediate intervention may benefit patients presenting emergently and may compensate for a weekend effect, but when care is delayed or pushed back until after the weekend, outcomes may be negatively impacted owing to more-severe disease presentation in the operating room. By contrast, a subgroup analysis demonstrated no difference in outcomes by case complexity (low vs high complexity). However, it is highly likely that surgeons may anticipate diminished access to resources over the weekend and intentionally schedule easier cases with less complex patients immediately before the weekend. The persistence of a weekend effect despite this potential mitigating bias lends further credence to the importance of understanding the weekend effect and its repercussions for patients. Finally, the weekend effect is diluted when expanding the preweekend and postweekend window, demonstrating that the association is greatest in the days immediately adjacent to the weekend.

Our findings underscore the need for a critical examination of current surgical scheduling practices and resource allocation. One approach for consideration is the optimization of perioperative care pathways to mitigate adverse outcomes. This may involve initiatives to improve adherence to standardized postoperative protocols, such as mobilization and Foley catheter removal,^24^ as well as enhanced communication strategies. Furthermore, systems-level approaches and health care policy efforts can also play a role in mitigating these disparities.

### Limitations

There are notable limitations to the study given its observational nature and use of administrative data. We do not have access to preoperative clinical data on patients and are, therefore, unable to stratify patients by immediate preoperative risk, which may be a potential confounder. Some patients had missing information and were, thus, eliminated from the cohort. In addition, we are unable to analyze the exact indications for complications and readmissions. Particularly for long-term outcomes, some adverse events may not be attributable to the index surgery. Some sensitivity analyses were conducted post hoc. In addition, we acknowledge the potential for ecological bias, as we are unable to assess for specific characteristics within subsets of our population that may influence the observed findings at the group level. Despite these shortcomings, the findings of this study may be used to guide health care decision-makers to address inefficiencies and better mitigate adverse outcomes brought on by the weekend effect.

## Conclusions

This study found a weekend effect across multiple surgical specialties, as evidenced by a small but significant increase in the risk of perioperative complications and long-term mortality of patients undergoing surgery immediately before the weekend. It is important for health care systems to assess how this phenomenon may impact their practices to ensure that patients receive excellent care irrespective of the day.

## Notes

Supplement 1. **eFigure**. Consort diagram **eTable 1.** Included procedures and corresponding OHIP fee codes **eTable 2.** Major postoperative complications and associated diagnostic and procedural codes **eTable 3.** Covariates and operationalized definitions **eTable 4.** Model fit and assessment of multicollinearity (VIF) **eTable 5.** Definitions of low- and high-complexity surgical procedures for subgroup analyses **eTable 6.** Surgeon and anesthesiologist years in practice and annual case volume in each day of the week **eTable 7.** Rate differences of composite endpoint after surgery administered 1-day pre- versus 1-day post-weekend **eTable 8.** Adjusted event rate or mean of outcomes within 30- and 90-days and 1-year of index surgery, by surgery in pre- or post-weekend using LSMEANS statement, excluding patients who underwent DES **eTable 9.** Sensitivity analyses: multivariable generalized estimating equation regression models, with clustering based on procedure fee code for outcomes within 30- and 90-days and 1-year of index surgery, by surgery in pre- or post-weekend **eTable 10.** Multivariable generalized estimating equation regression models, with clustering based on procedure fee code for outcomes within 30- and 90-days and 1-year of index surgery, by surgery in pre- or post-weekend **eTable 11.** Multivariable generalized estimating equation regression models, with clustering based on procedure fee code for outcomes within 30- and 90-days and 1-year of index surgery, by surgery performed on 2 days pre-weekend vs. 2 days post-weekend **eTable 12.** Multivariable generalized estimating equation regression models, with clustering based on procedure fee code for the composite outcome within 30- and 90-days and 1-year of index surgery, by surgery in pre- or post-weekend

Supplement 2.
Data Sharing Statement

## References

[1] McAlister FA, Youngson E, Padwal RS, Majumdar SR. Similar outcomes among general medicine patients discharged on weekends. J Hosp Med. 2015;10(2):69-74. doi:10.1002/jhm.2310 25537769

[2] Bell CM, Redelmeier DA. Waiting for urgent procedures on the weekend among emergently hospitalized patients. Am J Med. 2004;117(3):175-181. doi:10.1016/j.amjmed.2004.02.047 15276596

[3] Zapf MA, Kothari AN, Markossian T, . The “weekend effect” in urgent general operative procedures. Surgery. 2015;158(2):508-514. doi:10.1016/j.surg.2015.02.024 26013983 PMC5226376

[4] Zhou Y, Li W, Herath C, . Off-hour admission and mortality risk for 28 specific diseases: a systematic review and meta-analysis of 251 cohorts. J Am Heart Assoc. 2016;5(3):e003102. doi:10.1161/JAHA.115.003102 26994132 PMC4943279

[5] Freemantle N, Richardson M, Wood J, . Weekend hospitalization and additional risk of death: an analysis of inpatient data. J R Soc Med. 2012;105(2):74-84. doi:10.1258/jrsm.2012.120009 22307037 PMC3284293

[6] Barba R, Losa JE, Velasco M, Guijarro C, García de Casasola G, Zapatero A. Mortality among adult patients admitted to the hospital on weekends. Eur J Intern Med. 2006;17(5):322-324. doi:10.1016/j.ejim.2006.01.003 16864005

[7] Kang KM, Jeong KS, Oh HK, . The weekday effect on postoperative mortality in elective abdominal surgery: an observational study using propensity score methods. Surgery. 2021;170(1):186-193. doi:10.1016/j.surg.2020.12.033 33500156

[8] Zare MM, Itani KM, Schifftner TL, Henderson WG, Khuri SF. Mortality after nonemergent major surgery performed on Friday versus Monday through Wednesday. Ann Surg. 2007;246(5):866-874. doi:10.1097/SLA.0b013e3180cc2e60 17968181

[9] Aylin P, Alexandrescu R, Jen MH, Mayer EK, Bottle A. Day of week of procedure and 30 day mortality for elective surgery: retrospective analysis of hospital episode statistics. BMJ. 2013;346:f2424. doi:10.1136/bmj.f2424 23716356 PMC3665889

[10] Vail D, Pan C, Pershing S, Mruthyunjaya P. Association of rhegmatogenous retinal detachment and outcomes with the day of the week that patients undergo a repair or receive a diagnosis. JAMA Ophthalmol. 2020;138(2):156-163. doi:10.1001/jamaophthalmol.2019.5253 31855233 PMC6990708

[11] Al-Ashqar M, Aqil A, Phillips H, . There is no ‘weekend effect’ in elective orthopaedic surgery. Ann R Coll Surg Engl. 2018;100(7):551-555. doi:10.1308/rcsann.2018.0084 29909662 PMC6214053

[12] Sivaganesan A, Devin CJ, Khan I, . Is length of stay influenced by the weekday on which lumbar surgery is performed? Neurosurgery. 2019;85(4):494-499. doi:10.1093/neuros/nyy382 30165453

[13] Sedaghat AR, Metson R, Gray ST. Impact of day of week on outcomes of endoscopic sinus surgery for chronic rhinosinusitis. Am J Rhinol Allergy. 2015;29(5):378-382. doi:10.2500/ajra.2015.29.4228 26358351

[14] Satkunasivam R, Klaassen Z, Ravi B, . Relation between surgeon age and postoperative outcomes: a population-based cohort study. CMAJ. 2020;192(15):E385-E392. doi:10.1503/cmaj.190820 32392499 PMC7162435

[15] Wallis CJD, Jerath A, Coburn N, . Association of surgeon-patient sex concordance with postoperative outcomes. JAMA Surg. 2022;157(2):146-156. doi:10.1001/jamasurg.2021.6339 34878511 PMC8655669

[16] Wallis CJD, Jerath A, Kaneshwaran K, . Association between surgeon and anesthesiologist sex discordance and postoperative outcomes: a population-based cohort study. Ann Surg. 2022;276(1):81-87. doi:10.1097/SLA.0000000000005495 35703460

[17] von Elm E, Altman DG, Egger M, Pocock SJ, Gøtzsche PC, Vandenbroucke JP; STROBE Initiative. The Strengthening the Reporting of Observational Studies in Epidemiology (STROBE) statement: guidelines for reporting observational studies. Ann Intern Med. 2007;147(8):573-577. doi:10.7326/0003-4819-147-8-200710160-00010 17938396

[18] Govindarajan A, Urbach DR, Kumar M, . Outcomes of daytime procedures performed by attending surgeons after night work. N Engl J Med. 2015;373(9):845-853. doi:10.1056/NEJMsa1415994 26308685

[19] Urbach DR, Govindarajan A, Saskin R, Wilton AS, Baxter NN. Introduction of surgical safety checklists in Ontario, Canada. N Engl J Med. 2014;370(11):1029-1038. doi:10.1056/NEJMsa1308261 24620866

[20] Wallis CJ, Ravi B, Coburn N, Nam RK, Detsky AS, Satkunasivam R. Comparison of postoperative outcomes among patients treated by male and female surgeons: a population based matched cohort study. BMJ. 2017;359:j4366. doi:10.1136/bmj.j4366 29018008 PMC6284261

[21] Austin PC. Using the standardized difference to compare the prevalence of a binary variable between two groups in observational research. Commun Stat Simul Comput. 2009;38(6):1228-1234. doi:10.1080/03610910902859574

[22] Ruiz M, Bottle A, Aylin PP. Exploring the impact of consultants’ experience on hospital mortality by day of the week: a retrospective analysis of hospital episode statistics. BMJ Qual Saf. 2016;25(5):337-344. doi:10.1136/bmjqs-2015-004105 26202130

[23] Ruiz M, Bottle A, Aylin PP. The Global Comparators project: international comparison of 30-day in-hospital mortality by day of the week. BMJ Qual Saf. 2015;24(8):492-504. doi:10.1136/bmjqs-2014-003467 26150550 PMC4515980

[24] Liu JY, Merkow RP, Cohen ME, . Association of weekend effect with recovery after surgery. JAMA Surg. 2020;155(10):988-990. doi:10.1001/jamasurg.2020.2618 32845300 PMC7450403

[25] Smith SA, Yamamoto JM, Roberts DJ, . Weekend surgical care and postoperative mortality: a systematic review and meta-analysis of cohort studies. Med Care. 2018;56(2):121-129. doi:10.1097/MLR.0000000000000860 29251716 PMC5770102

[26] Palmer WL, Bottle A, Davie C, Vincent CA, Aylin P. Dying for the weekend: a retrospective cohort study on the association between day of hospital presentation and the quality and safety of stroke care. Arch Neurol. 2012;69(10):1296-1302. doi:10.1001/archneurol.2012.1030 22777008

[27] Shaheen AA, Kaplan GG, Myers RP. Weekend versus weekday admission and mortality from gastrointestinal hemorrhage caused by peptic ulcer disease. Clin Gastroenterol Hepatol. 2009;7(3):303-310. doi:10.1016/j.cgh.2008.08.033 18849015

[28] Dubois L, Vogt K, Vinden C, ; Surgical Investigators Group at ICES Western. Association between day of the week of elective surgery and postoperative mortality. CMAJ. 2017;189(8):E303-E309. doi:10.1503/cmaj.160511 27754897 PMC5325731

[29] Xie Y, Khanna S, Good N, Boyle J. Weekly hospital workforce data: a data visualisation exercise. Stud Health Technol Inform. 2017;239:153-159. doi:10.3233/978-1-61499-783-2-153 28756451

[30] Wong HJ, Morra D. Excellent hospital care for all: open and operating 24/7. J Gen Intern Med. 2011;26(9):1050-1052. doi:10.1007/s11606-011-1715-8 21499824 PMC3157523

[31] Becker DJ. Do hospitals provide lower quality care on weekends? Health Serv Res. 2007;42(4):1589-1612. doi:10.1111/j.1475-6773.2006.00663.x 17610439 PMC1955270

[32] Bailey HS, Mehrotra P, Drinkwater KJ, Howlett DC. National audit of seven-day working care in radiology. BJR Open. 2021;3(1):20200046. doi:10.1259/bjro.20200046 34381943 PMC8320131

[33] Varnava AM, Sedgwick JE, Deaner A, Ranjadayalan K, Timmis AD. Restricted weekend service inappropriately delays discharge after acute myocardial infarction. Heart. 2002;87(3):216-219. doi:10.1136/heart.87.3.216 11847156 PMC1767030

