# Supplementary material for: Postoperative Outcomes Following Preweekend Surgery
Source: JAMA Netw Open. 2025 Mar 4;8(3):e2458794. doi: 10.1001/jamanetworkopen.2024.58794 (PMC11880952; doi:10.1001/jamanetworkopen.2024.58794)
Supplement: Supplement 1. — eFigure. Consort diagram eTable 1. Included procedures and corresponding OHIP fee codes eTable 2. Major postoperative complications and associated diagnostic and procedural codes eTable 3. Covariates and operationalized definitions eTable 4. Model fit and assessment of multicollinearity (VIF) eTable 5. Definitions of low- and high-complexity surgical procedures for subgroup analyses eTable 6. Surgeon and anesthesiologist years in practice and annual case volume in each day of the week eTable 7. Rate differences of composite endpoint after surgery administered 1-day pre- versus 1-day post-weekend eTable 8. Adjusted event rate or mean of outcomes within 30- and 90-days and 1-year of index surgery, by surgery in pre- or post-weekend using LSMEANS statement, excluding patients who underwent DES eTable 9. Sensitivity analyses: multivariable generalized estimating equation regression models, with clustering based on procedure fee code for outcomes within 30- and 90-days and 1-year of index surgery, by surgery in pre- or post-weekend eTable 10. Multivariable generalized estimating equation regression models, with clustering based on procedure fee code for outcomes within 30- and 90-days and 1-year of index surgery, by surgery in pre- or post-weekend eTable 11. Multivariable generalized estimating equation regression models, with clustering based on procedure fee code for outcomes within 30- and 90-days and 1-year of index surgery, by surgery performed on 2 days pre-weekend vs. 2 days post-weekend eTable 12. Multivariable generalized estimating equation regression models, with clustering based on procedure fee code for the composite outcome within 30- and 90-days and 1-year of index surgery, by surgery in pre- or post-weekend [file jamanetwopen-e2458794-s001.pdf]

Supplemental Online Content

Ranganathan S, Riveros Sabogal C, Tsugawa Y, et al. Postoperative outcomes following preweekend surgery. *JAMA Netw Open*. 2025;8(2):e2458794. doi:10.1001/jamanetworkopen.2024.58794

eFigure. Consort diagram
eTable 1. Included procedures and corresponding OHIP fee codes
eTable 2. Major postoperative complications and associated diagnostic and procedural codes
eTable 3. Covariates and operationalized definitions
eTable 4. Model fit and assessment of multicollinearity (VIF)
eTable 5. Definitions of low- and high-complexity surgical procedures for subgroup analyses
eTable 6. Surgeon and anesthesiologist years in practice and annual case volume in each day of the week
eTable 7. Rate differences of composite endpoint after surgery administered 1-day pre- versus 1-day post-weekend
eTable 8. Adjusted event rate or mean of outcomes within 30- and 90-days and 1-year of index surgery, by surgery in pre- or post-weekend using LSMEANS statement, excluding patients who underwent DES
eTable 9. Sensitivity analyses: multivariable generalized estimating equation regression models, with clustering based on procedure fee code for outcomes within 30- and 90-days and 1-year of index surgery, by surgery in pre- or post-weekend
eTable 10. Multivariable generalized estimating equation regression models, with clustering based on procedure fee code for outcomes within 30- and 90-days and 1-year of index surgery, by surgery in pre- or post-weekend
eTable 11. Multivariable generalized estimating equation regression models, with clustering based on procedure fee code for outcomes within 30- and 90-days and 1-year of index surgery, by surgery performed on 2 days pre-weekend vs. 2 days post-weekend
eTable 12. Multivariable generalized estimating equation regression models, with clustering based on procedure fee code for the composite outcome within 30- and 90-days and 1-year of index surgery, by surgery in pre- or post-weekend

This supplemental material has been provided by the authors to give readers additional information about their work.

© 2025 Ranganathan S et al. *JAMA Network Open*.

eFigure. Consort Diagram

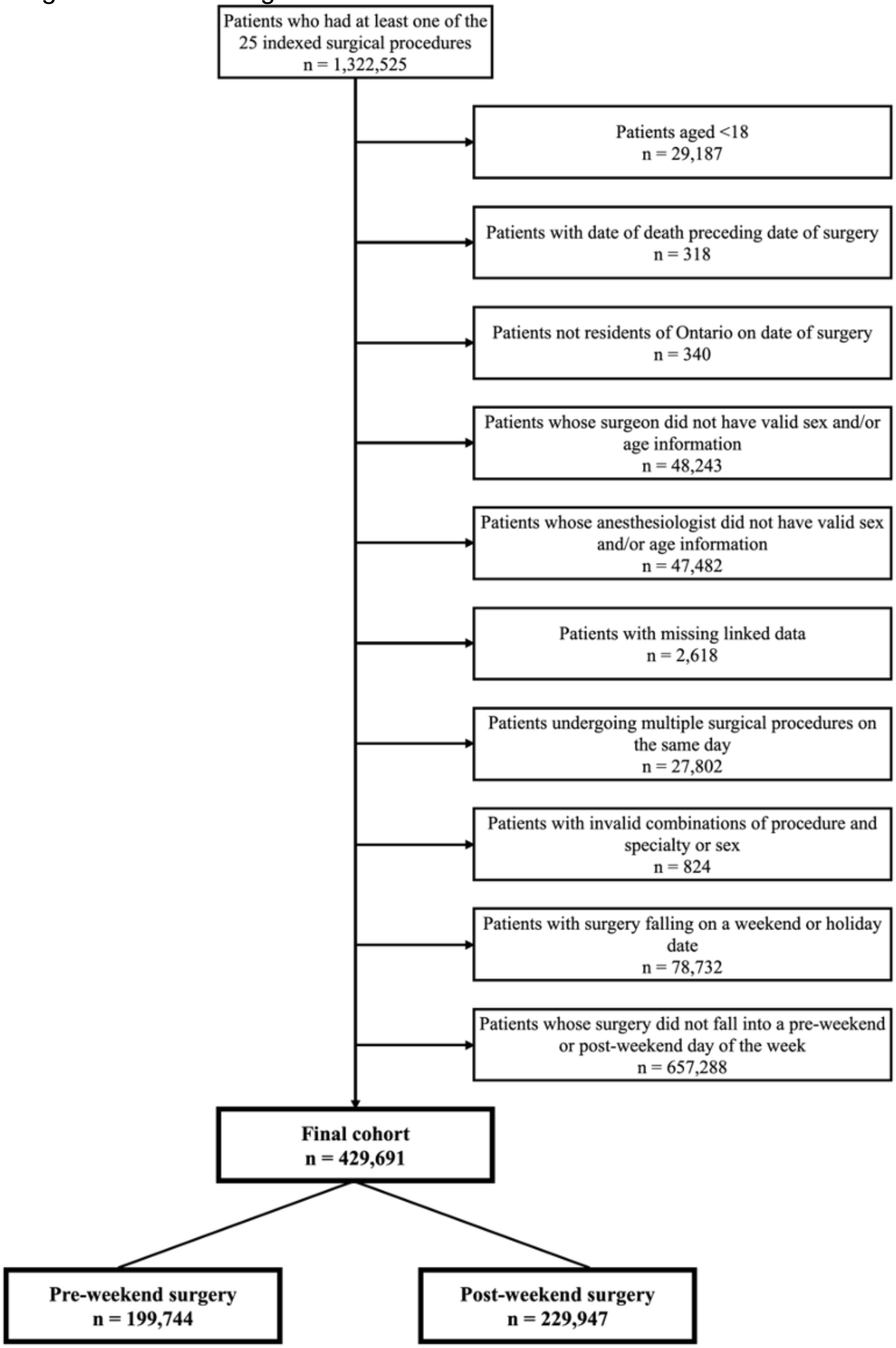

© 2025 Ranganathan S et al. *JAMA Network Open*.

eTable 1. Included procedures and corresponding OHIP fee codes

	Surgery	OHIP Fee Code
A.	Coronary artery bypass grafting	R742, R743
B.	Femoral-popliteal bypass	R791, R794
C.	Abdominal aortic aneurysm repair	R802, R817, R877
D.	Appendectomy	S205
E.	Cholecystectomy	S287
F.	Gastric bypass Roux-en-Y	S120
G.	Colon resection	S166, S167, S168, S169, S170, S171, S173, S174, S213, S214, S215, S216, S217
H.	Liver resection	S267, S269, S270, S271, S275
I.	Hysterectomy	S757, S816, S758, S759, S710, S763
J.	Spinal surgery	
i.	Anterior decompression	N500, N501, N502, N503, N504, N505, N506, N507, N508, N579
ii.	Anterior arthrodesis	N516, N517, N518, N559, N580
iii.	Posterior decompression	N509, N510, N520, N511, N512, N524
iv.	Posterior arthrodesis	N515, N519, N514, N581, N582, N533
K.	Craniotomy for brain tumor/biopsy	N102, N103, N151, N152, N153 / N113
L.	Knee replacement TKR	R441
M.	Hip replacement THR	R440
N.	Open repair femoral neck/femoral shaft fracture	F099, F100, F101/F096
O.	Total thyroidectomy	S788
P.	Neck dissection	R910, R915
Q.	Lung resection pneumonectomy, lobectomy or segmental resection	M142, M143, M144
R.	Radical cystectomy	S440, S453
S.	Radical prostatectomy	S651
T.	Transurethral resection of prostate	S655
U.	Carpal tunnel release	N290
V.	Breast reduction	R110

© 2025 Ranganathan S et al. *JAMA Network Open*.

eTable 2: Major postoperative complications and associated diagnostic and procedural codes.

Complication	ICD-10 diagnostic codes	CCI/ICD-10 procedure codes	OHIP fee codes
Acute renal failure	N17.x		
Bleeding requiring transfusion of 4 or more units within the first 72 hours after surgery	T81.0<br>T81.1<br>R58.x	1.LZ.19.^^	
Cardiac arrest requiring CPR	R09.2<br>I46.x		G395, G391, G521, G523, G522, G391
Coma ≥24 hours duration	R40.2x		
Deep venous thrombosis	I80.x		
Myocardial infarction	I21.x<br>I22.x<br>I23.x		
Ventilator use ≥48 hours	J95.1<br>J95.2<br>J95.3<br>J96.0		(G405 or G557) AND (G406 or G558)
Pneumonia	J12<br>J13<br>J14<br>J15<br>J16<br>J17<br>J18<br>J69.0		
Pulmonary embolism	I26.x		
Stroke	H34.1<br>I63.x<br>I64.x<br>I61.x<br>I60.x<br>G45.x		
Major disruption of wound	T81.3	1.SY.80.^^<br>(1 or more day after initial surgery)	S343
Infection of surgical site	T81.4		
Sepsis	A41.x		
Septic shock	A41.9		
Systemic inflammatory response syndrome	A41.x		

© 2025 Ranganathan S et al. *JAMA Network Open*.

Unplanned return to the operating room		1.OT.13.^^<br>1.OT.52.LA<br>1.OT.52.LA-TS<br>1.OT.70.LA<br>1.NK.76.^^<br>1.NK.77.^^<br>1.NK.80.^^<br>1.NK.87.^^<br>1.NM.52.^^<br>1.NM.76.^^<br>1.NM.77.^^<br>1.NM.80.^^<br>1.NM.87.^^<br>1.NM.89.^^<br>1.NM.91.^^<br>1.NP.86.^^	
Vascular graft failure	T82.3		
Shock	R57.x		

© 2025 Ranganathan S et al. *JAMA Network Open*.

eTable 3: Covariates and Operationalized Definitions

**Covariate**	
**Patient Data**	
Age at Index Surgery	0-99
Sex	Male
	Female
Socioeconomic Status Quintile*	Income quintile 1: Lowest quintile
	Income quintile 2: Medium-low quintile
	Income quintile 3: Middle quintile
	Income quintile 4: Medium-high quintile
	Income quintile 5: Highest quintile
Rurality	Urban
	Rural
LHIN	
Johns Hopkins Aggregate Diagnosis Group (ADG)	ADG 0-5
	ADG 6-7
	ADG 8-10
	ADG ≥11
**Surgeon Data**	
Age at Index Surgery	0-99
Sex	Male
	Female
Speciality	Cardiothoracic surgery
	General Surgery
	Neurosurgery
	Obstetrics and Gynaecology
	Orthopaedic Surgery
	Otolaryngology
	Plastic Surgery
	Thoracic Surgery
	Urology
	Vascular Surgery
Years in Practice	0-99
Annual volume of Index Procedure	Calculated by dividing the total number of patients seen in the previous year into quartiles
**Anesthesiologist Data**	
Age at Index Surgery	0-99
Sex	Male
	Female
Years in Practice	0-99
Annual case volume	Calculated by dividing the total number of patients seen in the previous year into quartiles
**Other characteristics**	
Hospital status	Community hospital
	Academic hospital
Surgical Procedure type	Elective

© 2025 Ranganathan S et al. *JAMA Network Open*.

	Urgent
Case Complexity	Low
	High
Duration of Surgery (minutes)	Missing duration
	Non-missing on duration, 0-999
Year of Index Surgery	2007-2019

*Socioeconomic Status:
Income quintile is based on neighborhood income quintile, based on patients' postal code.
From the Postal Code conversion file plus (PCCF+) version 8a, reference Guide by StatCan:
2021 Census Profile data available at the dissemination area (DA) geographic level were used to calculate neighborhood income quintiles and deciles. This analysis relied on single-person equivalent (SPE) to take into consideration household size. SPE is the person-weights implicit in the Statistics Canada low-income cut-offs needed to specific an appropriate multiplier for different household sizes. This adjustment is necessary since it generally costs less per person for tow or more persons living together compared to one person living alone. SPE values were based on low-income lines for 2020.

For each DA, total income was calculated by multiplying the median income (either before- or after-tax) by the number of households. That total income (for each DA) was then divided by the sum of single-person equivalent in the DA to obtain income per single person equivalent (IPSPEs). In the 2021 Statistics Canada census, there were a total of 57,936 DAs. Complete income and population data were available for 54,191 DAs. The IPSPEs for 3,262 DAs were imputed based on the average IPSPE values in adjacent DAs (as defined using a first-order queen spatial adjaceny matrix). 483 DAs had missing income data as well as no adjacent DAs that could be used for imputation.

National-level neighborhood income quintiles and deciles were constructed by ranking all DAs in Canade from the lowest IPSPE to the highest IPSPE, and then assigning each DA to one of five groups (for quintiles) and one of ten groups (for deciles). The assignment of DAs to national-level income quintiles and deciles was weighted by residential population, such that each group contained approximately one-fifth or one-tenth of the total in-scope population.

In addition to national-level quantiles, area-based income quintiles and deciles were created based on the set of DAs within each census metropolitan area (CMA), each census agglomeration (CA), and for the residual non-CMA and non-CA DAs within each province. To be clear, each DA was assigned into one geographical group; a CMA (if the DA was located in a CMA), a CA (if the DA was located in a tracted CA), or a province/territory (if the DA was located outside of a CMA or tracted CA). There were 63 geographical groups used to calculate area-based income quintiles and deciles (i.e. 63 different quintile and decile rankings). Area-based income quintiles and deciles were constructed by ranking all DAs within a geographical group from the lowest IPSPE to the highest IPSPE, and then assigning each DA to one of five groups (for quintiles) and to one of ten groups (for deciles). The assignement of DAs to area-based income quintiles and deciles was weighted by residential population, such that each group contrained approximately one-fifth or one-tenth of the total in-scope population within a CMA, a CA, or the non-CMA and non-CA DAs in a province. The rationale for creating area-based income quintiles is that housing and other costs vary across Canada. For example, rents and house prices in some places, such as most of Quebec and the Atlantic provinces, have historyically been lower than those in Toronta, Ontario, or Vancouver, British Columbia.

© 2025 Ranganathan S et al. *JAMA Network Open*.

eTable 4: Model Fit and Assessment of Multicollinearity (VIF)

GEE Model Fit Statistics			
**Outcome**		**QIC**	**QICu**
**30 days**	Composite Outcome	303495.1914	301323.3298
	Death	24504.7465	24011.386
	Readmission	164046.2001	163386.2058
	Complications	193556.7849	191604.8991
	LOS	-1509134.837	-1529194.495
	Index Surgery Duration	-121931189.7	-121950039.8
**90 days**	Composite Outcome	378228.645	375616.6732
	Death	52502.829	51173.0372
	Readmission	248775.5533	247513.9405
	Complications	201480.1947	199543.232
	LOS	-1852553.95	-1863906.495
**1 year**	Composite Outcome	630893.9395	627455.4479
	Death	125028.2473	121277.7168
	Readmission	511047.3853	507421.1026
	Complications	234694.6288	232888.8728
	LOS	-2382611.912	-2389162.139

Assessment of Multicollinearity (VIF)

Variable	VIF	Tolerance (1/VIF)
**flag_1dwkend**	1.0102	0.9899
**surg_age**	3.4445	0.2903
**surg_sex_1**	1.2225	0.8180
**surg_specname_1**	65.4417	0.0153
**surg_specname_2**	15.6347	0.0640
**surg_specname_3**	37.1384	0.0269
**surg_specname_4**	66.5220	0.0150
**surg_specname_5**	6.3897	0.1565
**surg_specname_6**	13.1819	0.0759
**surg_specname_7**	4.9416	0.2024
**surg_specname_8**	21.3477	0.0468
**surg_specname_9**	1.9259	0.5192
**surg_volgrp_1**	1.6245	0.6156
**surg_volgrp_2**	1.7138	0.5835
**surg_volgrp_3**	1.9685	0.5080
**surg_yrpract**	3.4602	0.2890
**ane_age**	3.4390	0.2908
**ane_sex_1**	1.0363	0.9649
**ane_volgrp_1**	1.7035	0.5870
**ane_volgrp_2**	1.8813	0.5315
**ane_volgrp_3**	2.1650	0.4619
**ane_yrpract_cor**	3.4244	0.2920
**age**	1.4073	0.7106
**sex_1**	1.3263	0.7540

© 2025 Ranganathan S et al. *JAMA Network Open*.

**pat_adggrp_1**	1.4808	0.6753
**pat_adggrp_2**	1.5638	0.6395
**pat_adggrp_3**	1.5052	0.6644
**bl_inc_1**	1.6543	0.6045
**bl_inc_2**	1.6644	0.6008
**bl_inc_3**	1.6827	0.5943
**bl_inc_4**	1.6794	0.5955
**bl_rural**	1.1887	0.8413
**lhin_1**	2.4116	0.4147
**lhin_2**	2.0250	0.4938
**lhin_3**	2.9248	0.3419
**lhin_4**	1.8647	0.5363
**lhin_5**	2.2513	0.4442
**lhin_6**	2.2043	0.4537
**lhin_7**	2.7819	0.3595
**lhin_8**	2.7674	0.3613
**lhin_9**	1.8171	0.5503
**lhin_10**	2.6651	0.3752
**lhin_11**	1.5897	0.6290
**lhin_12**	1.9400	0.5155
**lhin_13**	1.3762	0.7266
**idx_surg_teach**	1.5369	0.6507
**index_year_1**	1.8373	0.5443
**index_year_2**	1.8484	0.5410
**index_year_3**	1.8388	0.5438
**index_year_4**	1.8329	0.5456
**index_year_5**	1.8171	0.5503
**index_year_6**	1.8485	0.5410
**index_year_7**	1.8464	0.5416
**index_year_8**	1.8293	0.5466
**index_year_9**	1.7780	0.5624
**index_year_10**	1.7132	0.5837
**index_year_11**	1.6530	0.6050
**index_year_12**	1.6027	0.6240
**Mean VIF**	**5.6443**	

© 2025 Ranganathan S et al. *JAMA Network Open*.

eTable 5: Definitions of low- and high-complexity surgical procedures for subgroup analyses

Category	Low complexity procedures	High complexity procedures
**Definition**	procedures with little variability from case to case (“straightforward” procedures) and those which are performed by a significant proportion of surgeons within that specialty	Remaining procedures not categorized as low complexity
**Included procedures:**	appendectomy, cholecystectomy, carpal tunnel release	coronary artery bypass grafting, femoral-popliteal bypass, abdominal aortic aneurysm repair, gastric bypass, colon resection, liver resection, anterior or posterior spinal decompression, anterior or posterior spinal arthrodesis, craniotomy for brain tumor, total knee replacement, total hip replacement, open repair of femoral neck or shaft fracture, total thyroidectomy, neck dissection, lung resection, radical cystectomy

© 2025 Ranganathan S et al. *JAMA Network Open*.

eTable 6: Surgeon and Anesthesiologist Years in Practice and Annual Case Volume in Each Day of the Week

Group	Variable	Label	Mon-Fri<br>N=429,691	Monday<br>N=205,814	Tuesday<br>N=22,622	Wednesday<br>N=1,981	Thursday<br>N=6,924	Friday<br>N=192,350	P value
Surgeon	Years in practice	Mean (SD)	23.1 (10.0)	23.4 (9.9)	24.8 (10.1)	23.3 (9.9)	24.1 (10.0)	22.5 (10.2)	<0.001
		Median (IQR)	22.2 (14.8-30.6)	22.6 (15.4-30.7)	24.5 (16.6-32.9)	23.0 (15.0-30.5)	23.8 (15.8-31.8)	21.3 (14.0-30.2)	<0.001
	Annual case volume								
	1st Quartile	no. (%)	101,562 (23.6%)	47,244 (46.5%)	4,627 (4.6%)	536 (0.5%)	1,563 (1.5%)	47,592 (46.9%)	
	2nd Quartile	no. (%)	110,381 (25.7%)	53,904 (48.8%)	5,518 (5.0%)	513 (0.5%)	1,818 (1.6%)	48,628 (44.1%)	
	3rd Quartile	no. (%)	108,278 (25.2%)	53,798 (49.7%)	5,697 (5.3%)	448 (0.4%)	1,770 (1.6%)	46,565 (43.0%)	
	4th Quartile	no. (%)	109,470 (25.5%)	50,868 (46.5%)	6,780 (6.2%)	484 (0.4%)	1,773 (1.6%)	49,565 (45.3%)	<0.001
Anesthesiologist	Years in practice	Mean (SD)	23.4 (10.7)	23.4 (10.7)	23.7 (10.7)	23.4 (10.8)	23.6 (10.5)	23.3 (10.8)	<0.001
		Median (IQR)	22.7 (14.4-31.7)	22.8 (14.6-31.6)	23.2 (14.9-31.9)	23.0 (14.0-31.0)	22.8 (14.8-31.8)	22.6 (14.2-31.7)	<0.001
	Annual case volume								
	1st Quartile	no. (%)	99,630 (23.2%)	48,424 (48.6%)	5,030 (5.0%)	497 (0.5%)	1,592 (1.6%)	44,087 (44.3%)	
	2nd Quartile	no. (%)	111,807 (26.0%)	53,230 (47.6%)	5,608 (5.0%)	542 (0.5%)	1,875 (1.7%)	50,552 (45.2%)	
	3rd Quartile	no. (%)	108,873 (25.3%)	51,457 (47.3%)	5,849 (5.4%)	521 (0.5%)	1,753 (1.6%)	49,293 (45.3%)	
	4th Quartile	no. (%)	109,381 (25.5%)	52,703 (48.2%)	6,135 (5.6%)	421 (0.4%)	1,704 (1.6%)	48,418 (44.3%)	<0.001

Note: Percentages are row % in each day of the week.

© 2025 Ranganathan S et al. *JAMA Network Open*.

eTable 7: Rate difference of composite endpoint after surgery administered 1-day pre- versus 1-day post-weekend. 95% Confidence Intervals (CI) were obtained by using the 2.5% and 97.5% percentiles through 1,000 bootstrap replications

Outcome	Rate Difference	Lower 95% CI	Upper 95% CI
Composite endpoint at 30 days	0.36	0.21	0.49
Composite endpoint at 90 days	0.57	0.39	0.74
Composite endpoint at 1 year	0.81	0.58	1.04

© 2025 Ranganathan S et al. *JAMA Network Open*.

eTable 8: Adjusted Event Rate or Mean of Outcomes within 30- and 90-Days and 1-Year of Index Surgery, by Surgery in Pre- or Post-Weekend using LSMEANS Statement, excluding Patients who Underwent DES

Outcome	Label	Time Period for Outcome	Pre-Weekend Adjusted Rate or Mean (95% CI)	Post-Weekend Adjusted Rate or Mean (95% CI)
Composite Endpoint	Rate (%)	Within 30 Days	8.24 (7.38-9.19)	7.91 (7.07-8.84)
Death	Rate (%)		0.13 (0.08-0.20)	0.12 (0.08-0.19)
Re-admission	Rate (%)		4.21 (3.85-4.62)	4.06 (3.72-4.44)
Complications	Rate (%)		4.05 (3.42-4.80)	3.91 (3.28-4.65)
Hospital Stay (days)	Mean		3.34 (3.05-3.67)	3.16 (2.90-3.46)
Duration of Index Surgery (minutes)	Mean		143.57 (130.19-158.32)	142.70 (129.61-157.11)
Composite Endpoint	Rate (%)	Within 90 Days	11.79 (10.96-12.68)	11.26 (10.50-12.08)
Death	Rate (%)		0.31 (0.21-0.47)	0.28 (0.18-0.44)
Re-admission	Rate (%)		7.65 (7.01-8.34)	7.29 (6.70-7.94)
Complications	Rate (%)		4.34 (3.70-5.09)	4.19 (3.55-4.93)
Hospital Stay (days)	Mean		3.96 (3.60-4.35)	3.71 (3.40-4.05)
Composite Endpoint	Rate (%)	Within 1 Year	22.16 (20.59-23.85)	21.41 (20.08-22.83)
Death	Rate (%)		1.27 (0.91-1.78)	1.15 (0.81-1.64)
Re-admission	Rate (%)		17.72 (15.85-19.81)	17.19 (15.53-19.04)
Complications	Rate (%)		5.55 (4.93-6.24)	5.37 (4.74-6.07)
Hospital Stay (days)	Mean		5.39 (4.90-5.92)	5.07 (4.66-5.52)

Note: using GEE modeling dealing with clustering based on procedure fee code (Poisson distribution with log link for binary outcomes and Negative Binominal with log link for continuous outcomes, using LSMEAN 13tatement to obtain adjusted rates or means), adjusted for surgeon age, surgeon sex, surgeon annual case volume, surgeon years of practice, anesthesiologist age, anesthesiologist sex, anesthesiologist annual case volume, anesthesiologist years of practice, patient age, patient sex, patient comorbidity, rurality, income quintile , index year, and hospital status.

© 2025 Ranganathan S et al. *JAMA Network Open*.

eTable 9. Sensitivity Analysis: Multivariable Generalized Estimating Equation Regression Models, with Clustering based on Procedure Fee Code for Outcomes within 30- and 90-Days and 1-Year of Index Surgery, by Surgery in Pre- or Post-Weekend

	**Outcome within 30 Days**		**Outcome within 90 Days**		**Outcome within 1 Year**	
**Outcome**	**Pre- vs. Post-Weekend aOR/aRR (95% CI)**	**P value**	**Pre- vs. Post-Weekend aOR/aRR (95% CI)**	**P value**	**Pre- vs. Post-Weekend aOR/aRR (95% CI)**	**P value**
Composite Endpoint	1.05 (1.03-1.08)	<0.001	1.06 (1.03-1.09)	<0.001	1.06 (1.03-1.09)	<0.001
* Adjusted odds ratio (aOR) for binary outcomes and adjusted relative risk (aRR) for continuous outcomes.						
Note: using GEE modeling dealing with clustering based on procedure fee code (logistic regression with binomial distribution and logit link), adjusted for surgeon age (continuous), surgeon sex, surgeon annual case volume (quartiles), surgeon specialty, surgeon years of practice (continuous), anesthesiologist age (continuous), anesthesiologist sex, anesthesiologist annual case volume (quartiles), anesthesiologist years of practice (continuous), patient age (continuous), patient sex, patient comorbidity (categorical), rurality (rural vs. urban), income quintile, LHIN, hospital status (academic vs. community), and index year, **as well as duration of index surgery.**						

© 2025 Ranganathan S et al. *JAMA Network Open*.

eTable 10. Multivariable Generalized Estimating Equation Regression Models, with Clustering based on Procedure Fee Code for Outcomes within 30- and 90-Days and 1-Year of Index Surgery, by Surgery in Pre- or Post-Weekend

	**Outcome within 30 Days**		**Outcome within 90 Days**		**Outcome within 1 Year**	
**Outcome**	**Pre- vs. Post-Weekend aOR/aRR (95% CI)**	**P value**	**Pre- vs. Post-Weekend aOR/aRR (95% CI)**	**P value**	**Pre- vs. Post-Weekend aOR/aRR (95% CI)**	**P value**
Composite Endpoint	1.05 (1.02-1.08)	<0.001	1.06 (1.03-1.09)	<0.001	1.05 (1.02-1.09)	<0.001
Death	1.09 (1.03-1.16)	0.004	1.10 (1.03-1.17)	0.003	1.12 (1.08-1.17)	<0.001
Re-admission	1.04 (1.01-1.06)	0.01	1.05 (1.03-1.08)	<0.001	1.04 (1.01-1.07)	0.002
Complications	1.05 (1.01-1.09)	0.006	1.05 (1.01-1.09)	0.007	1.05 (1.02-1.08)	0.003
Hospital Stay (days)	1.06 (1.04-1.08)	<0.001	1.07 (1.05-1.09)	<0.001	1.07 (1.05-1.10)	<0.001
Duration of Index Surgery	1.01 (0.99-1.03)	0.54	n.a		n.a	

* Adjusted odds ratio (aOR) for binary outcomes and adjusted relative risk (aRR) for continuous outcomes.

Note: using GEE modeling dealing with clustering based on procedure fee code (logistic regression with binomial distribution and logit link for binary outcomes, and negative binomial distribution and log link for continuous outcomes), adjusted for surgeon age (continuous), surgeon sex, surgeon annual case volume (quartiles), surgeon specialty, surgeon years of practice (continuous), anesthesiologist age (continuous), anesthesiologist sex, anesthesiologist annual case volume (quartiles), anesthesiologist years of practice (continuous), patient age (continuous), patient sex, patient comorbidity (categorical), rurality (rural vs. urban), income quintile, LHIN, hospital status (academic vs. community), and index year.

© 2025 Ranganathan S et al. *JAMA Network Open*.

eTable 11. Multivariable Generalized Estimating Equation Regression Models, with Clustering based on Procedure Fee Code for Outcomes within 30- and 90-Days and 1-Year of Index Surgery, by Surgery Performed on 2 days pre-weekend vs. 2 days post-weekend

		Outcome within 30 Days		Outcome within 90 Days		Outcome within 1 Year	
Model No.	Outcome	Friday vs. Monday aOR/aRR (95% CI)	P value	Friday vs. Monday aOR/aRR (95% CI)	P value	Friday vs. Monday aOR/aRR (95% CI)	P value
1	Composite Endpoint	1.03 (1.01-1.05)	0.006	1.04 (1.02-1.06)	<0.001	1.03 (1.01-1.05)	0.004
2	Death	1.03 (0.99-1.08)	0.14	1.04 (0.98-1.10)	0.18	1.08 (1.04-1.12)	<0.001
3	Re-admission	1.02 (1.00-1.05)	0.09	1.03 (1.02-1.05)	<0.001	1.02 (1.01-1.04)	0.006
4	Complications	1.03 (1.00-1.05)	0.02	1.02 (1.00-1.05)	0.04	1.02 (1.00-1.04)	0.06
5	Hospital Stay (days)	1.03 (1.02-1.05)	<0.001	1.04 (1.02-1.06)	<0.001	1.04 (1.03-1.06)	<0.001
6	Duration of Index Surgery	0.99 (0.98-1.01)	0.46	n.a		n.a	

* Adjusted odds ratio (aOR) for binary outcomes and adjusted relative risk (aRR) for continuous outcomes.

Note: using GEE modeling dealing with clustering based on procedure fee code (logistic regression with binomial distribution and logit link for binary outcomes, and negative binomial distribution and log link for continuous outcomes), adjusted for surgeon age (continuous), surgeon sex, surgeon annual case volume (quartiles), surgeon specialty, surgeon years of practice (continuous), anesthesiologist age (continuous), anesthesiologist sex, anesthesiologist annual case volume (quartiles), anesthesiologist years of practice (continuous), patient age (continuous), patient sex, patient comorbidity (categorical), rurality (rural vs. urban), income quintile, LHIN, hospital status (academic vs. community), and index year.

© 2025 Ranganathan S et al. *JAMA Network Open*.

eTable 12. Multivariable Generalized Estimating Equation Regression Models, with Clustering based on Procedure Fee Code for the Composite Outcome within 30- and 90-Days and 1-Year of Index Surgery, by Surgery in Pre- or Post-Weekend based on Adjustments for Patient, Facility, and Surgeon Characteristics

		Outcome within 30 days		Outcome within 90 days		Outcome within 1 year	
**Analysis**	**Outcome**	**Pre- vs. Post-Weekend Crude OR/aOR (95% CI)**	**P value**	**Pre- vs. Post-Weekend Crude OR/aOR (95% CI)**	**P value**	**Pre- vs. Post-Weekend Crude OR/aOR (95% CI)**	**P value**
Unadjusted Analysis	Composite Endpoint	1.12 (1.05-1.18)	0.0001	1.11 (1.05-1.17)	0.0001	1.08 (1.03-1.13)	0.0030
Adjusted for patient and facility factors	Composite Endpoint	1.11 (1.05-1.17)	0.0002	1.10 (1.05-1.16)	0.0001	1.08 (1.03-1.12)	0.0003
Adjusted for patient, facility, and physician factors	Composite Endpoint	1.03 (1.01-1.05)	0.0057	1.04 (1.02-1.06)	0.0002	1.03 (1.01-1.05)	0.0036

© 2025 Ranganathan S et al. *JAMA Network Open*.
