# Supplementary material for: Postoperative Outcomes Following Preweekend Surgery
Source: JAMA Netw Open. 2025 Mar 4;8(3):e2458794. doi: 10.1001/jamanetworkopen.2024.58794 (PMC11880952; doi:10.1001/jamanetworkopen.2024.58794)
Supplement: Supplement 2. — Data Sharing Statement [file jamanetwopen-e2458794-s002.pdf]

Data Sharing Statement

Ranganathan. Postoperative Outcomes Following Preweekend Surgery. *JAMA Netw Open*. Published February 20, 2025. doi:10.1001/jamanetworkopen.2024.58794

Data
Data available: No
